# Intrauterine hyperglycaemia during late gestation caused mitochondrial dysfunction in skeletal muscle of male offspring through CREB/PGC1A signaling

**DOI:** 10.1038/s41387-024-00299-x

**Published:** 2024-07-23

**Authors:** Yi-Shang Yan, Jia-Ying Mo, Yu-Tong Huang, Hong Zhu, Hai-Yan Wu, Zhong-Liang Lin, Rui Liu, Xuan-Qi Liu, Ping-Ping Lv, Chun Feng, Jian-Zhong Sheng, Min Jin, He-Feng Huang

**Affiliations:** 1 The Second Affiliated Hospital, School of Medicine, Zhejiang University, Hangzhou, China; 2 Key Laboratory of Reproductive Genetics, Ministry of Education, Zhejiang University, Hangzhou, China; 3 The Fourth Affiliated Hospital, School of Medicine, Zhejiang University, Yiwu, China; 4 The Obstetrics & Gynecology Hospital of Fudan University, Shanghai, China

**Keywords:** Nutrition disorders, Endocrine system and metabolic diseases

## Abstract

**Background:**

Maternal diabetes mellitus can influence the development of offspring. Gestational diabetes mellitus (GDM) creates a short-term intrauterine hyperglycaemic environment in offspring, leading to glucose intolerance in later life, but the long-term effects and specific mechanism involved in skeletal muscle dysfunction in offspring remain to be clarified.

**Methods:**

Pregnant mice were divided into two groups: The GDM group was intraperitoneally injected with 100 mg/kg streptozotocin on gestational days (GDs) 6.5 and 12.5, while the control (CTR) group was treated with vehicle buffer. Only pregnant mice whose random blood glucose level was higher than 16.8 mmol/L beginning on GD13.5 were regarded as the GDM group. The growth of the offspring was monitored, and the glucose tolerance test was performed at different time points. Body composition analysis and immunohistochemical methods were used to evaluate the development of lean mass at 8 weeks. The exercise capacity and grip strength of the male mouse offspring were assessed at the same period. Transmission electron microscopy was used to observe the morphology inside skeletal muscle at 8 weeks and as a foetus. The genes and proteins associated with mitochondrial biogenesis and oxidative metabolism were investigated. We also coanalyzed RNA sequencing and proteomics data to explore the underlying mechanism. Chromatin immunoprecipitation and bisulfite-converted DNA methylation detection were performed to evaluate this phenomenon.

**Results:**

Short-term intrauterine hyperglycaemia inhibited the growth and reduced the lean mass of male offspring, leading to decreased endurance exercise capacity. The myofiber composition of the tibialis anterior muscle of GDM male offspring became more glycolytic and less oxidative. The morphology and function of mitochondria in the skeletal muscle of GDM male offspring were destroyed, and coanalysis of RNA sequencing and proteomics of foetal skeletal muscle showed that mitochondrial elements and lipid oxidation were consistently impaired. In vivo and in vitro myoblast experiments also demonstrated that high glucose concentrations impeded mitochondrial organisation and function. Importantly, the transcription of genes associated with mitochondrial biogenesis and oxidative metabolism decreased at 8 weeks and during the foetal period. We predicted *Ppargc1α* as a key upstream regulator with the help of IPA software. The proteins and mRNA levels of *Ppargc1α* in the skeletal muscle of GDM male offspring were decreased as a foetus (CTR vs. GDM, 1.004 vs. 0.665, *p* = 0.002), at 6 weeks (1.018 vs. 0.511, *p* = 0.023) and 8 weeks (1.006 vs. 0.596, *p* = 0.018). In addition, CREB phosphorylation was inhibited in GDM group, with fewer activated pCREB proteins binding to the CRE element of *Ppargc1α* (1.042 vs. 0.681, *p* = 0.037), *Pck1* (1.091 vs. 0.432, *p* = 0.014) and *G6pc* (1.118 vs. 0.472, *p* = 0.027), resulting in their decreased transcription. Interestingly, we found that sarcopenia and mitochondrial dysfunction could even be inherited by the next generation.

**Conclusions:**

Short-term intrauterine hyperglycaemia significantly reduced lean mass in male offspring at 8 weeks, resulting in decreased exercise endurance and metabolic disorders. Disrupted organisation and function of the mitochondria in skeletal muscle were also observed among them. Foetal exposure to hyperglycaemia decreased the ratio of phosphorylated CREB and reduced the transcription of *Ppargc1α*, which inhibited the transcription of downstream genes involving in mitochondrial biogenesis and oxidative metabolism. Abnormal mitochondria, which might be transmitted through aberrant gametes, were also observed in the F2 generation.

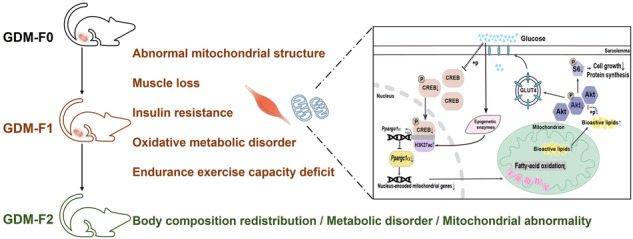

## Introduction

The “Foetal Origins of Adult Disease” hypothesis is an emerging theory that indicates that intrauterine malnutrition could have adverse effects on the development and function of tissues later in life [1]. Gestational diabetes mellitus (GDM) is a common gestational complication that is characterised by abnormal glucose tolerance during the third trimester and recovery to normal levels after birth [2]. Glucose is known to cross the placenta into the foetal circulation. Evidence suggests that short-term intrauterine hyperglycaemia could negatively affect foetal development and islet function [3], brown adipose tissue (BAT) [4] and the liver [5, 6], leading to metabolic syndrome in adulthood.

Skeletal muscle (SKM) dysfunction plays an important role in obesity, insulin resistance (IR) and diabetes [7]. One study demonstrated that intrauterine hyperglycaemia could result in insulin resistance in offspring SKMs at three weeks old [8]. Clinical investigations revealed that the IR offspring of T2DM parents exhibited impaired mitochondrial activity in SKM [9]. Maternal metabolic syndrome was demonstrated to cause mitochondrial dysfunction in F2 SKM via aberrant oocyte mitochondria in F1 females [10]. However, whether and how GDM affects SKM development in offspring are still unclear.

The pivotal component of mitochondrial biogenesis, peroxisome proliferator-activated receptor-gamma coactivator-1 alpha (*Ppargc1α*), has attracted increased amounts of attention as a marker of muscle wasting [11]. Work from our group has demonstrated that intrauterine hyperglycaemia inhibited the expression of *Ppargc1α* and uncoupling protein (*Ucp1*) in BAT, leading to impaired mitochondrial thermogenesis [4]. We wondered whether the expression of *Ppargc1α* in GDM skeletal muscle was altered and what the potential mechanism was involved. Moreover, we were also curious about whether the adverse effects of GDM on skeletal muscle could be transmitted to the next generation.

In summary, we demonstrated that short-term foetal exposure to hyperglycaemia downregulated *Ppargc1α* by inhibiting CREB phosphorylation in the SKM of male offspring, leading to abnormal mitochondrial biogenesis and lipid oxidation. Thus, GDM male offspring developed muscle loss, a decrease in exercise endurance capacity and metabolic dysfunction at 8 weeks. Moreover, F1 foetal exposure to hyperglycaemia contributed to adult F2 metabolic disorders, obesity, and mitochondrial abnormalities in skeletal muscle.

## Materials and methods

### Animals

Institute of Cancer Research (ICR) mice aged 8 weeks were purchased from Shanghai SLAC Laboratory Animal Co. (Shanghai, China). The method for establishing the GDM model was modified from previous work [12]. Virgin female mice were mated with male mice. Once the vaginal plug was observed, the day was considered gestational day 0.5 (GD0.5) for the mother and embryonic day 0.5 (ED0.5) for the foetus. Time-pregnant mice were assigned to the control (CTR) or GDM group. Each GDM female was treated with 100 mg/kg streptozotocin (Sigma‒Aldrich, St. Louis, MO, USA; S0130) dissolved in citrate buffer (Beijing Solarbio Science & Technology Co., Ltd., China; C1013) at GD6.5 and GD12.5 after overnight fasting. CTR mice received an equal volume of buffer. Blood glucose was detected daily from GD13.5 via the tail vein. GDM was defined only when the random glucose level was higher than 16.8 mmol/L. The offspring of GDM were breastfed by normal female mice for 3 weeks.

### Physiological measurements and metabolic testing

After weaning, the weights of the offspring were recorded weekly. Intraperitoneal glucose (2 g/kg body weight) tolerance tests (GTTs) and insulin (0.8 U/kg body weight) resistance tests (ITTs) were performed after 16-h and 6-h fasts, respectively. In vivo oxygen consumption and carbon dioxide emission were measured over 24 h in a TSE LabMaster System (TSE Systems, Bad Homburg, Germany) when the animals had access to food and water. The body composition of the conscious mice was detected through a nuclear magnetic resonance instrument (Niumag Corporation, China, QMR06-090H). Gripping force was evaluated by a grip strength metre (Xin Run Corporation, China, XR501): the mouse was held by the tail and placed on the net. Then, we pulled the mice gently back by the tail so that their forepaws grabbed the net, and the grip in Newtons (N) was recorded until the net was released. There was no acclimation period for the test. The process was repeated three times, the highest force was recorded and we calculated the average. The exercise endurance capacity (EEC) test was performed as follows: Mice were acclimated to the device for 4 consecutive days for 10 min at a speed of 15 meter per minute (m/min). The mice were exercised at the start of the dark cycle, such as at 5:00 pm. The speeds were adjusted using following protocol:15 m/min for 45 min, 18 m/min for 15 min, 20 m/min for 15 min, 22 m/min for 15 min and 25 m/min for up to 60 min. Mice were constantly monitored and were motivated to exercise via an electric stimulus of 0.5 mA. Exhaustion was considered after 6 s of permanence on the electric grid, and the mouse did not run anymore even if it was tapped. Maximum exercise capacity was estimated using parameters: the duration of the run (min) and the distance (m).

### Immunohistochemical and immunofluorescence staining

The frozen skeletal muscle was cut into 8 millimetre (mm) thick cross sections without fixation. The Tetrazole Salt Method was used according to the kit protocol (Solarbio Science & Technology Co., Ltd., China, G2000) to stain for succinate dehydrogenase (SDH) inside the muscle. 8 mm-thick-sections were fixed with 4% paraformaldehyde, and antigen retrieval was performed accordingly (Beyotime Biotech. Inc., China, P0090). After they were incubated in 0.05% Triton X-100 (Thermo Fisher Scientific, USA, BP151-500) and washed with PBS, the sections were incubated with primary antibodies overnight at 4 °C followed by secondary antibodies (Supplementary Table 1) for 1 h at room temperature. Images were observed and captured with an Olympus IX83-FV3000-OSR. More than six sections were randomly selected to estimate the myofiber cross-sectional area (CSA) and distribution using image analysis software (Image-Pro Plus, Media Cybernetics, Silver Spring, MD, USA).

### Transmission electron microscopy (TEM)

Skeletal muscle was minced into small pieces and fixed in 2.5% glutaraldehyde at 4 °C overnight. After being fixed in 1% osmium tetroxide for 1 h and stained with uranyl acetate for half an hour, the muscle minces were gradient dehydrated. Then, the muscle was embedded and stained with uranyl acetate and lead citrate. Images were acquired by a 120 kV cryo-transmission electron microscope (Thermo FEI, Czech, Tecnai G2 spirit).

### RNA sequencing and proteomics

Foetal limb muscle was extracted after the skin and bones were removed. Total RNA was isolated and purified using TRIzol reagent (TaKaRa, Japan, 9108) following the manufacturer’s procedure. The RNA concentration and purity of each sample were quantified using a NanoDrop ND-1000 (NanoDrop, Wilmington, DE, USA). An RNA-Seq library preparation kit was used to generate cDNA. Finally, we performed 2 × 150 bp paired-end sequencing (PE150) on an Illumina NovaSeq™ 6000 (LC-Bio Technology Co., Ltd., Hangzhou, China). After removing low-quality and undetermined bases, HISAT2 software was used to map the reads to the genome (*Mus musculus*, Ensembl v101). The total differentially expressed genes are listed in Data Set 1. Compared to those in the CTR group, the significant differentially expressed genes (DEGs) with a fold change ≥2 or ≤0.5 and a *P* ≤ 0.05 were regarded as significant. The DEGs were further subjected to prediction of potential upstream regulators via Ingenuity Pathway Analysis (IPA; QIAGEN, Valencia, CA, USA). Muscle protein was prepared by SDT lysis and homogenised. After labelling using TMT reagent (Thermo Fisher Scientific), liquid chromatography-tandem mass spectrometry (LC-MS/MS) analysis was performed on a Q Exactive plus mass spectrometer coupled to an Easy nLC, and the raw files were processed using the MASCOT engine (Matrix Science, London, UK; version 2.6) embedded into Proteome Discoverer 2.2. The total differentially expressed proteins are listed in Data Set 2. Significant differentially expressed proteins (DEPs) between groups were defined on the basis of a fold change ≥1.2 or ≤0.833 with a *P* ≤ 0.05. Histograms and bubble diagrams were generated with OmicStudio at http://www.omicstudio.cn/tool.

### Isolation of primary myoblasts from skeletal muscle

Myoblasts were isolated as previously reported [13]. Briefly, skeletal muscle was removed, washed, and minced into small pieces. Then, collagenase II (Worthington Biochemical, USA, LS004176, final concentration 400 U/ml) was added to the media for digestion in a 15 ml tube. The tube was shaken at 37 °C at most speeds for 1 h with another 5 s vortex. The tubes were spun at 1400 × *g* for 5 min, after which the supernatant was discarded. The pellet was resuspended in media and pipetted several times using a sterile 10 ml pipette. The resuspended mixture was collected and passed through prewet 70 and 30 μm strainers. The tubes were spun at 1400 × *g* for 5 min. The pellet was resuspended in DMEM (American Type Culture Collection, USA, 30-2002) and seeded onto a 6-cm dish. After 24 h, the supernatant was collected and spun at 930 × *g* for 5 min, after which the pellet was resuspended in DMEM supplemented with bFGF (Thermo Fisher Scientific, USA, 100-18B), after which the mixture was transferred to 10% Matrigel-coated dishes (Corning, Inc., USA, 354234).

### In vivo and in vitro myoblast experiments

The oxygen consumption rate (OCR) was measured with a Seahorse XFe96 Analyser (Agilent Technologies, USA). Briefly, cells were seeded on culture microplates (Agilent Technologies, 101085-004) the day before. The following mitochondrial respiratory inhibitors were injected: oligomycin (Merck SA, Germany; 75351, final concentration in the well: 1 μM), fluorocarbon cyanide phenylhydrazone (Merck SA; C2920, final concentration in the well: 1 μM), rotenone (Merck SA; R8875, final concentration in the well: 1 μM) and antimycin A (Merck SA; A8674, final concentration in the well: 5 μM). The basal respiration, adenosine triphosphate (ATP) production, maximal respiration, proton leakage and spare capacity were calculated.

Specific small interfering RNAs (siRNAs) targeting mouse *Ppargc1α* (RiboBio Co., Ltd., Guangzhou, China; siB12322140811) were purchased. C2C12 myoblast at 30–40% confluency was transfected with 50 nM siRNA using Lipofectamine™ RNAiMAX (Thermo Fisher Scientific, Inc., 13778150) according to the manufacturer’s protocol.

When C2C12 cells (ATCC® CRL-1772™, RRID: CVCL_0188) were grown in DMEM (Biological Industries, Israel, 06-1055-57-1ACS) supplemented with 10% (v/v) foetal bovine serum (Biological Industries, Israel, 04-001-1ACS) to 80–90% confluence, pECMV-Ppargc1α-m-FLAG (MiaoLingBio, China, P8854) and pECMV-MCS-FLAG (MiaoLingBio, China, P0787) were obtained, and 2.5 μg plasmids were transfected with Lipofectamine 3000 (Thermo Fisher Scientific, Inc., L3000015) according to the manufacturer’s protocol. The transfected cells were harvested after 48 h.

H89 (MedChemExpress, HY-15979) and Forskolin (MedChemExpress, HY-15371) were dissolved in dimethyl sulfoxide (DMSO), diluted to 50 μM in medium and cultured for another 48 h.

C2C12 cells and myoblasts were cultured in DMEM supplemented with low glucose (HyClone, USA; SH30021.01) or high glucose (HyClone, USA; SH30243.01) when necessary. High glucose media contained D-glucose at a concentration of 4500 mg/L, and the D-glucose concentration was 1000 mg/L in low glucose media.

### Real-time quantitative RT‒PCR

Total RNA was isolated from skeletal muscle of different ages and from myoblasts by using TRIzol (Takara, Otsu, Shiga, Japan, 9108). Total RNA (at most 1 mg) was reverse transcribed to cDNA with a PrimeScript RT Reagent Kit (Takara, RR036A). TB Green Premix Ex Taq (Takara, RR420A) was used for real-time quantitative PCR. The relative quantification of each mRNA was calculated using Actin or 18S as an internal reference. The primers used are listed in Supplementary Table 2.

### Western blot

Referring to the phosphorylation status of the insulin pathway, every male mouse aged 8 weeks was intraperitoneally injected with 0.08 U/kg insulin after fasting for 6 h. Fifteen minutes after the key proteins in the pathway were fully activated by insulin, the mice were sacrificed, and limb muscles, specifically the quadriceps femoris (QUA), tibialis anterior (TA), gastrocnemius (GAS) and soleus (SOL) muscles, were collected. Whole limb muscle was collected when it referred to the foetal experiment. A total of 15–30 mg of skeletal muscle protein was separated using 10% polyacrylamide gels and then transferred to a polyvinylidene fluoride membrane. The membranes were incubated with the antibodies listed in Supplementary Table 1. The bands were visualised by a chemiluminescence system and was quantified by ImageJ software.

### DNA methylation

Genomic DNA was collected from foetal limb muscle using the TIANamp Genomic DNA Kit (TIANGEN Biotech Co., Ltd., Beijing, China, DP304). Bisulfite was converted using the EpiTect Fast DNA Bisulfite Kit (Qiagen, 59802). The primers were designed with Qiagen PyroMark Assay Design 2.0 software and covered specific CpG islands in the target genes (Supplementary Table 2). Purified converted DNA was amplified with a PyroMark PCR Kit. Pyrosequencing was conducted on a pyrosequencer (Qiagen, PyroMark Q24).

### Chromatin immunoprecipitation (ChIP)

Each immunoprecipitation assay was performed using fresh foetal limb muscle according to the protocol of the SimpleChIP® Plus Enzymatic Chromatin IP Kit (Magnetic Beads) (Cell Signaling Technology (CST), Danvers, Massachusetts, USA, 9005). The samples were first fixed with 1.5% formaldehyde for crosslinking for 20 min at room temperature, after which the reaction was terminated by the addition of glycine. The compact tissues were isolated into cell suspensions using Dounce homogenizers. Micrococcal nuclease (CST, 10011) was added together with ultrasonication (Diagenode, Belgium, Bioruptor Pico) to digest DNA to approximately 150–900 bp in length, followed by EDTA (CST, 7011) to stop digestion. Ten microlitres of a total 500 μl of chromatin sample were used for a 2% input control. Primary antibody or anti-IgG (CST, 2729) 1.5 μg was added to 200 μl of chromatin sample, which was subsequently incubated with rotation overnight at 4 °C. 25 μl of Protein G Magnetic Beads (CST,9006) was added to each IP and input reaction, and the mixture was rotated at 4 °C for another 2 h. DNA was ultimately purified after elution from the chromatin-antibody-bead mixture. The primers used are listed in Supplementary Table 2. ChIP sequencing was conducted by Novogene (Beijing, China). The data were processed through the Integrative Genomics Viewer (IGV).

### Statistical analysis

The data are shown as the mean ± SEM. In the F1 generation, the differences in body composition, muscle weight ratio, area under the curve (AUC) of GTT and ITT, grip force, gene expression, and western blot data between the CTR and GDM groups were evaluated by a two-tailed Student’s *t* test. GTTs, ITTs, and weight monitoring in F1 generation were generally analysed via two-way ANOVA. The results were subsequently compared according to the time points in detail by Sidak’s multiple comparisons test. In the F2 generation, body composition, muscle weight ratio, the area under the curve (AUC) of the GTT and ITT, and gene expression were compared among the four groups by one-way ANOVA. Weight monitoring, GTT, and ITT were performed through two-way ANOVA, generally followed by Tukey’s multiple comparisons test, specifically at the time points. A *P* < 0.05 was considered to indicate statistical significance. GraphPad Prism software (GraphPad Software, San Diego, CA, USA, version 8.3.1) was utilised for statistical analysis and graphics.

## Results

### Short-term intrauterine hyperglycaemia led to a decrease in endurance capacity and systematic metabolic disorders in F1 adult mice

**Fig. 1:**
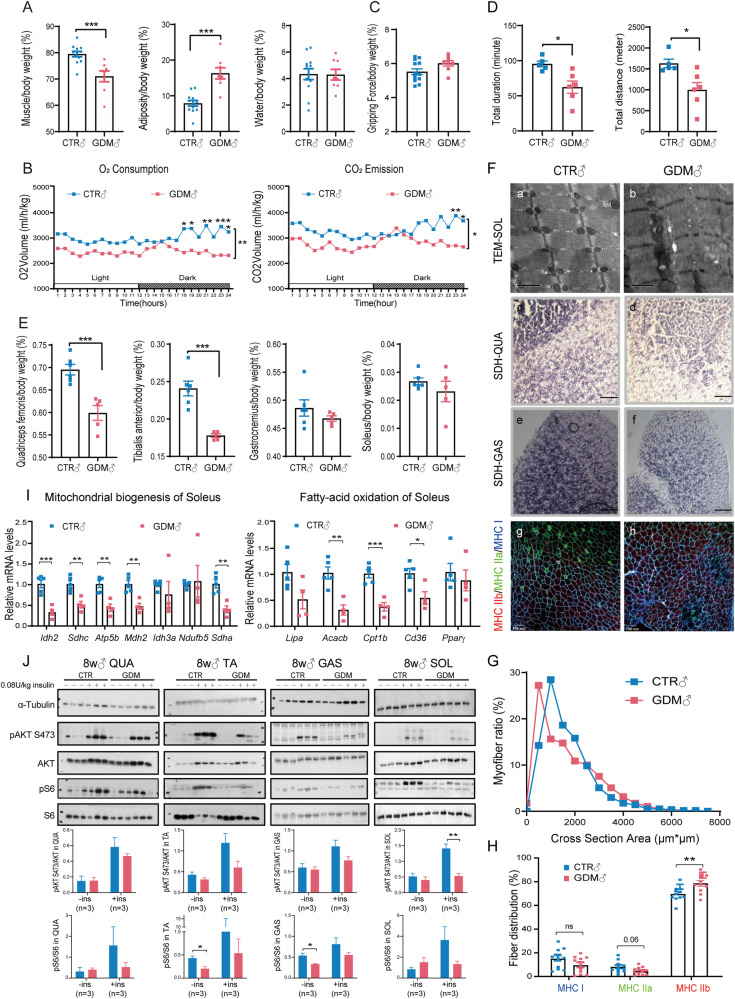
Intrauterine hyperglycaemia during late gestation leads to sarcopenia and causes mitochondrial abnormalities and insulin resistance in skeletal muscle in F1 males at 8 weeks. **A** GDM male offspring were composed of more adiposity and less muscle (n_CTR_= 12, n_GDM_ = 8, each “n” refers to a single mouse, two-tailed *t* test); **B** GDM male offspring consumed less oxygen and produced less carbon dioxide (*n* = 8, 2-way ANOVA); **C** No difference in gripping force between two groups was found (n_CTR_= 13, n_GDM_ = 8, two-tailed *t* test); **D** Endurance exercise capacity test was demonstrated to be poor in GDM male offspring (n_CTR_= 5, n_GDM_ = 6, two-tailed *t* test); **E** QUA and TA weights normalised to body weight were significantly lighter in GDM male offspring (n_CTR_= 6, n_GDM_ = 5, two-tailed *t* test); **F** TEM of SOL (a, b) showed swollen mitochondria with vacuoles, SDH staining of QUA (c, d) and GAS (e, f) revealed smaller staining area in GDM male offspring. Immunofluorescence of TA (g, h) showed composition of specific metabolic type of myofibers; **G** CSA of TA in GDM male offspring was smaller (*n* = 15); H: Myofiber type distribution according to immunofluorescent staining in TA showed glycolytic myofibers (MHCIIb) significantly increased, while oxidative myofibers (MHCI and MHCIIa) tended to decrease (*n* = 11, multiple *t* tests); **I** Genes related to mitochondrial biogenesis and oxidative metabolism by RT-qPCR were found to be inhibited in GDM male offspring soleus (n_CTR_= 5, n_GDM_ = 4, multiple *t* tests); **J** Representative western blotting images showed insulin signalling proteins were inhibited to be phosphorylated in GDM male offspring muscle at basal and insulin stimulation (*n* = 3, multiple *t* tests); Data are expressed as the mean ± SEM. Significance of the differences: *p < 0.05, **p < 0.01, ***p < 0.001.

To test the impact of short-term intrauterine hyperglycaemic exposure on growth, we first monitored weight gain in F1 generation for a long time. The body weight was lower in GDM offspring of both sexes (Supplementary Figs. 1A, 2A), although it presented a trend towards catch-up growth. Through a glucose tolerance test, GDM offspring were shown to have systematic metabolic disorders. Exposure to intrauterine hyperglycaemia resulted in glucose intolerance as early as 8 weeks in male mice (Supplementary Fig. 1B, C). GDM females also exhibited severe damage in glucose metabolism at 12 weeks, which displayed a delay compared to males (Supplementary Fig. 2B, C). Body composition analysis of 8-week-old mice demonstrated that GDM male offspring were composed of more adiposity and had a lower muscle mass ratio (Fig. 1A), which might cause less oxygen consumption and carbon dioxide emission (Fig. 1B). We did not find a difference in grip strength between the two groups (Fig. 1C). However, the total duration and distance GDM mice ran were significantly reduced (Fig. 1D). We then picked four pieces of skeletal muscle for further research. The percentages of quadriceps femoris (QUA) and tibialis anterior (TA) muscles were obviously lower in the GDM male group. Neither the gastrocnemius (GAS) nor the soleus (SOL) region reached statistical significance (Fig. 1E). Concerning the aspects mentioned above, discrepancies in female offspring at 8 weeks were modest (Supplementary Fig. 2D–F).

### Short-term intrauterine hyperglycaemia caused mitochondrial abnormalities and insulin resistance in the skeletal muscle of F1 males

We observed oxidative myofiber distribution and mitochondrial morphology inside male skeletal muscle at 8 weeks. TEM revealed that GDM male mice had fewer mitochondria in the soleus and that there were vacuoles in swollen mitochondria (Fig. 1Fa, b). Histological analysis of QUA and GAS also revealed a smaller area of SDH staining, which indicated mitochondrial abnormalities (Fig. 1Fc–f). We performed immunofluorescence staining of specific metabolic types of myofibers in the TA (Fig. 1Fg, h) and evaluated the CSA and distribution of different types of myofibers. We found that the CSA of GDM tended to be smaller (Fig. 1G). The number of glycolytic myofibers stained with MHCIIb significantly increased, while the number of oxidative myofibers stained with MHCI and MHCIIa tended to decrease in the GDM group but not significantly (Fig. 1H). We investigated whether this phenotype was related to oxidative disorders and mitochondrial biogenesis by RT-qPCR. Notably, the expression of genes involved in oxidative metabolism and the electron transport chain, including *Idh2*, *Sdhc*, *Atp5b*, *Mdh2*, *Sdha*, *Acacb*, *Cpt1b* and *Cd36*, significantly decreased in the GDM soleus population (Fig. 1I). In addition to the soleus, the transcription of genes in the same series was verified to be inhibited in QUA and GAS at 8 weeks (Supplementary Fig. 1D–I). The expression of metabolic genes was also verified to be suppressed to some extent in the GDM male soleus at 6 weeks (Supplementary Fig. 1J). However, we did not find any significant difference in gene expression among GDM females at 12 weeks (Supplementary Fig. 2G). To determine whether mitochondrial dysfunction in the muscle of GDM males was associated with a poor response to insulin, we used western blotting to test the classical insulin pathway. According to our analytical data, compared to the GDM males, the levels of phosphorylated S6 in the TA and GAS were significantly greater in the CTR group after fasting for 4 h before insulin stimulation. Under insulin stimulation, phosphorylated AKT protein only in the SOL was dramatically greater in CTR males than in GDM males, while the phosphorylation of AKT and S6 in other muscle tended to be inhibited even though not significantly in GDM group (Fig. 1J). These data suggested that mitochondrial dysfunction in GDM male SKM at 8 weeks was associated with a poor response to insulin and might lead to systematic glucose intolerance and insulin resistance.

### Mitochondrial biogenesis and oxidative metabolism were impaired in foetal GDM male skeletal muscle

**Fig. 2:**
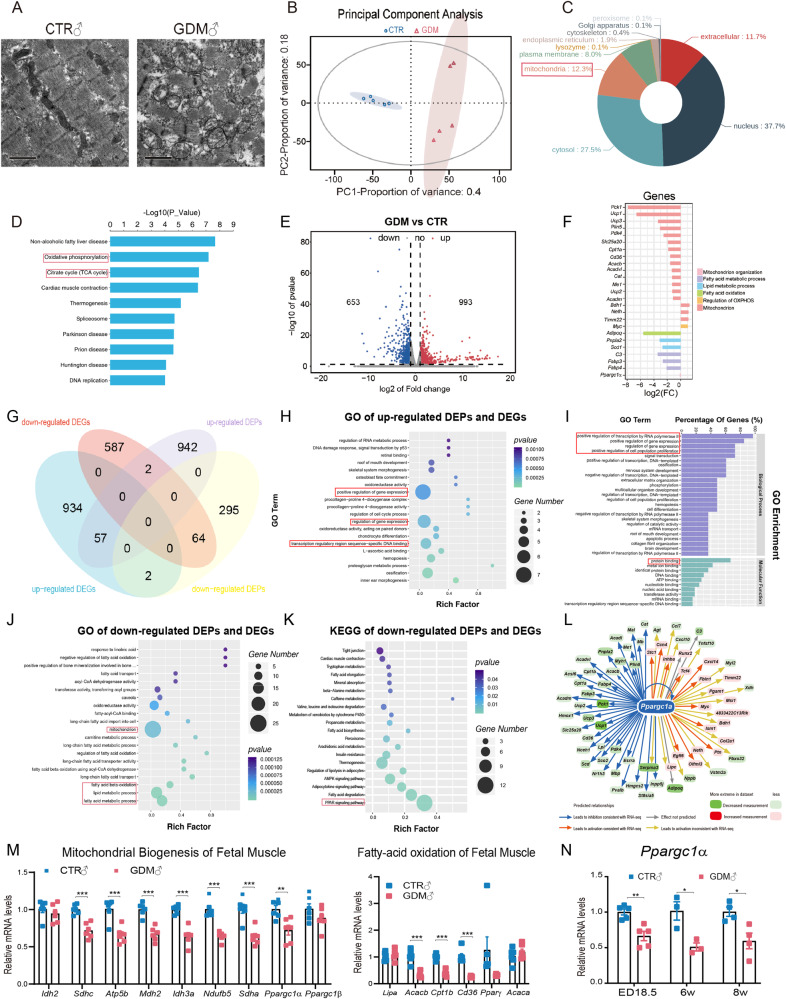
RNA-seq and proteomics analysis of foetal skeletal muscle and verification. **A** TEM showed myofiber and mitochondrial malformation in foetal GDM lower limb skeletal muscle; **B** Principal component analysis of proteomics verified that protein sample between groups were sufficiently heterogeneous; **C** DEPs were mainly distributed in nucleus, cytosol and mitochondria; **D** KEGG analysis of DEPs showed OXPHOS and TCA cycle were highly enriched; **E** Volcano plot of DEGs showed 993 upregulated and 653 downregulated genes were identified as significance; **F** Histogram of gene transcription levels related to mitochondria and metabolic processes according to RNA-seq; **G** Venn diagram of coanalysis of DEGs in RNA-seq and DEPs in proteomics; **H**, **I** GO analysis showed both upregulated DEPs and DEGs were mainly enriched in positive gene expression and transcription; **J** GO analysis showed both downregulated DEPs and DEGs were mainly enriched in mitochondrion and fatty-acid or lipid metabolic process; **K** KEGG analysis of both downregulated DEPs and DEGs were mainly focused on PPAR signalling and thermogenesis; **L** IPA software predicted that *Ppargc1α* inhibition was the key regulator of mitochondrial and oxidative disorder; **M** RT-qPCR showed representative mRNA levels related to mitochondrial biogenesis and fatty acid oxidation in foetal muscles were largely inhibited in GDM male offspring (*n* = 5, multiple *t* tests); **N** RT-qPCR showed mRNA levels of *Ppargc1α* decreased at ED18.5 (*n* = 5), 6 weeks (*n* = 3) and 8 weeks (*n* = 4) in GDM male offspring by multiple *t* tests; Data are expressed as the mean ± SEM. Significance of the differences: *p < 0.05, **p < 0.01, ***p < 0.001.

After exploring the effect of GDM on foetal skeletal muscle, we found that foetal skeletal muscle not only displayed muscle malformation but also mitochondrial abnormalities (Fig. 2A). After that, we performed proteomic analyses of lower limb muscle tissue at ED18.5. Principal component analysis (PCA) of the proteomic data showed no significant difference in the CTR group, but the protein samples between the groups were heterogeneous (Fig. 2B). DEPs were mainly spread across the nucleus (37.7%), cytosol (27.5%) and mitochondria (12.3%) (Fig. 2C). We then performed Kyoto Encyclopedia of Genes and Genomes (KEGG) enrichment analysis and found that the enriched pathways involved oxidative phosphorylation (OXPHOS) and the citrate cycle (Fig. 2D). At the same time, a total of 993 upregulated and 653 downregulated genes were identified via RNA-seq (Fig. 2E). The transcription of specific genes related to metabolic processes and mitochondria according to RNA-seq was mostly inhibited (Fig. 2F). We then coanalyzed the DEGs and DEPs, and the Venn diagram showed 57 upregulated genes at both the RNA and protein levels (Fig. 2G). The Gene Ontology (GO) term showed that these genes were enriched mainly in the positive regulation of gene expression and transcription (Fig. 2H, I). Sixty-four downregulated genes overlapped at the RNA and protein levels (Data Set 3). GO analysis revealed these genes related to mitochondrion and lipid or fatty acid metabolism (Fig. 2J). KEGG analysis also revealed that PPAR signalling was the top signalling pathway affected (Fig. 2K). Next, we performed IPA and demonstrated that *Ppargc1α* might be an upstream regulator of DEGs (Fig. 2L). RT-qPCR confirmed that *Sdhc, Atp5b, Mdh2, Sdha, Acacb, Cpt1b,* and *Cd36* were indeed downregulated in the muscles at both 8 weeks (Fig. 1I) and at ED18.5 (Fig. 2M), which was consistent with the sequencing results. We tested genes related to mitochondrial dynamics and copies in foetal muscle, but the results showed no difference (Supplementary Fig. 1K, L). RT-qPCR revealed that the expression of *Myf5* and *Pax3*, which are markers of myoblasts, were upregulated by intrauterine hyperglycaemia (Supplementary Fig. 1M). We also confirmed that the mRNA level of *Ppargc1α* was continuously downregulated at ED18.5, 6 weeks, and 8 weeks (Fig. 2N). Disrupted mitochondrial structure was also observed in the muscles of GDM female foetuses (Supplementary Fig. 2H). However, the transcription of genes related to mitochondrial biogenesis and oxidative metabolism was not significantly altered (Supplementary Fig. 2I). The statistical analysis revealed that mitochondrial dysfunction in GDM adult SKM resulted from abnormal mitochondrial biogenesis in its foetus.

### *Ppargc1α* knockdown led to impaired mitochondrial oxidative metabolism

**Fig. 3:**
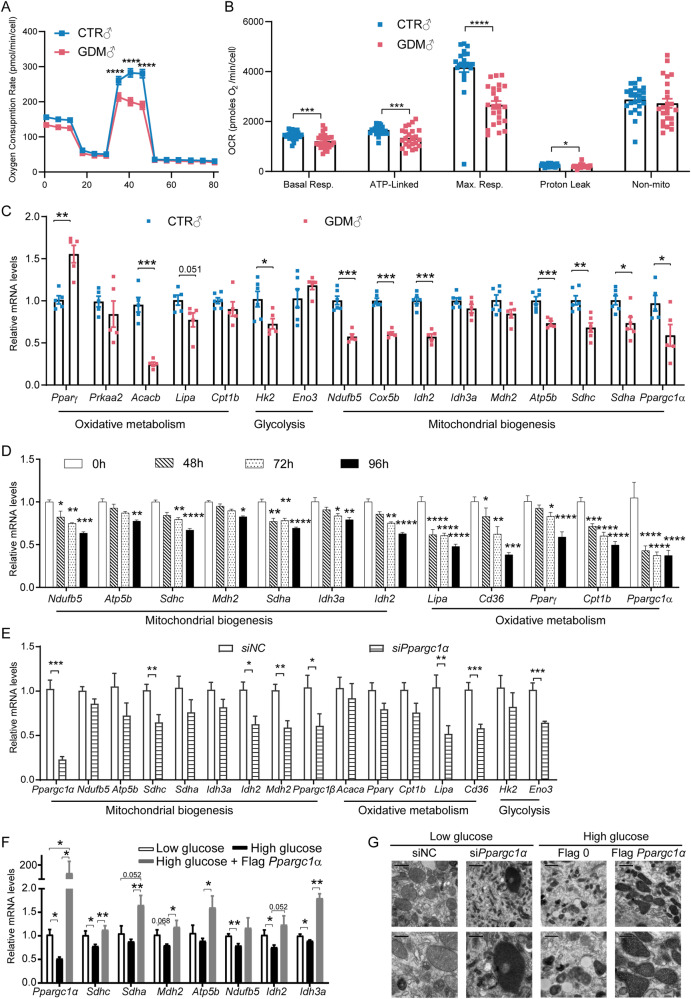
In vivo and in vitro experiments in myoblasts demonstrated that high glucose concentrations impaired mitochondrial transcription and function by inhibiting *Ppargc1α* transcription. **A** Oxygen consumption rate (OCR) was decreased in GDM male myoblast (*n* = 8, 2-way ANOVA); **B** Analysis of the OCR produced during basal respiration, ATP-linked process, maximal respiration, proton leakage and nonmitochondrial oxidation showed only OCR in mitochondria was decreased in GDM male myoblast (*n* = 24, multiple *t* tests); **C** Representative mRNA levels of genes related to mitochondrial biogenesis and metabolism were also inhibited in GDM foetal isolated primary myoblasts determined by RT-qPCR (*n* = 5, multiple *t* tests); **D** Representative mRNA levels of genes mentioned above were time-dependently down-regulated on C2C12 incubation in high glucose (*n* = 3, multiple *t* tests); **E** Representative mRNA levels of genes mentioned above were lower in C2C12 after *Ppargc1α* silencing (*n* = 6, multiple *t* tests); **F** Representative mRNA levels of genes mentioned above were higher in C2C12 after *Ppargc1α* overexpression (*n* = 5, multiple *t* tests); **G** TEM showed *Ppargc1α* positively regulated the formation of mitochondria in C2C12 myoblasts. The data are expressed as the mean ± SEM. Significance of the differences: *p < 0.05, **p < 0.01, ***p < 0.001, ****p < 0.0001.

We extracted undifferentiated myoblasts from the foetus. Both male (Fig. 3A) and female (Supplementary Fig. 2J) myoblasts presented a decrease in oxygen consumption, especially in mitochondria (Fig. 3B). The expression of mitochondrial oxidative metabolism-related genes in GDM primary myoblasts was also downregulated (Fig. 3C). We then incubated C2C12 myoblasts in high-glucose medium for different durations. The RT-qPCR results showed that the longer cells were cultured in high glucose, the greater mitochondrial oxidation was inhibited (Fig. 3D). As IPA software predicted *Ppargc1α* as a regulator of down-regulated mitochondrial genes, we performed *Ppargc1α* silencing in C2C12 cells and found it restrained mitochondrial biogenesis and oxidative metabolism (Fig. 3E). However, overexpression of *Ppargc1α* promoted the transcription of these genes (Fig. 3F). TEM images revealed a decrease in mitochondria and inner mitochondrial structural disorders after *Ppargc1α* knockdown, while more mitochondria and cristae were clearly observed when *Ppargc1α* was overexpressed (Fig. 3G). We also assessed whether mitochondrial dynamics changed according to changes in *Ppargc1α* levels: overexpressing or knocking out *Ppargc1α* did not influence mitochondrial fusion and fission directly (Supplementary Fig. 1O, P), which was the same as what was observed in foetal muscle (Supplementary Fig. 1K). These results indicated that intrauterine hyperglycaemia during late gestation downregulated *Ppargc1α,* causing mitochondrial malformation and dysfunction in foetal muscle.

### Inhibiting the transcription of *Ppargc1α* by decreasing CREB phosphorylation resulted in mitochondrial dysfunction in the muscle of GDM male foetuses

**Fig. 4:**
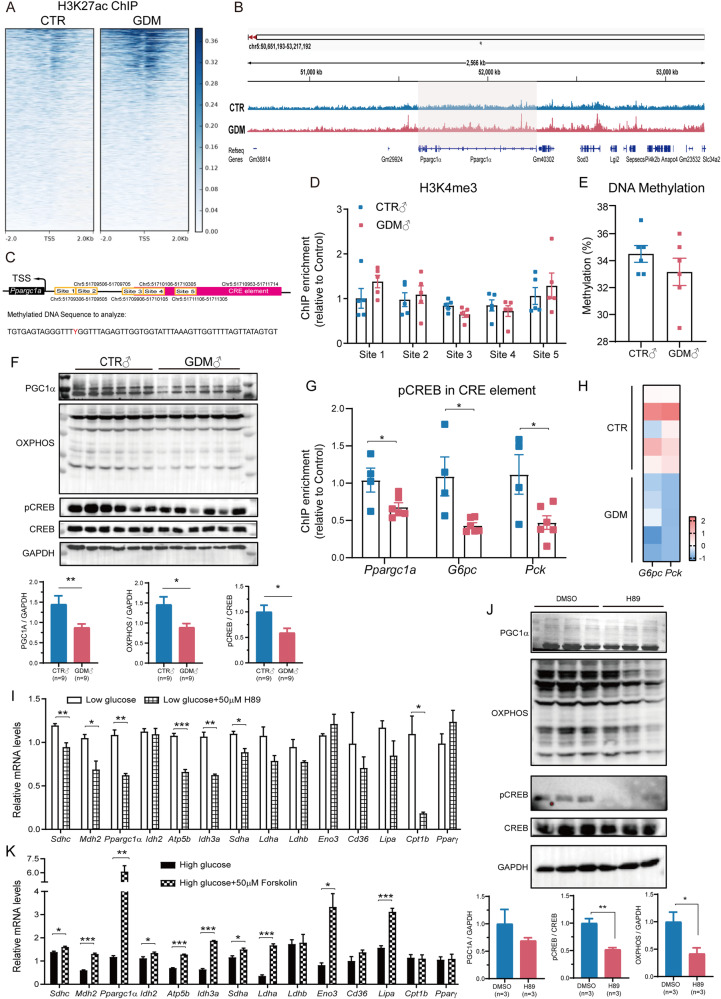
Intrauterine hyperglycaemia inhibited the expression of *Ppargc1α* in foetal skeletal muscle through CREB/PGC1A signalling. **A** ChIP sequence heatmap of genome-wide binding of H3K27ac showed that H3K27ac were highly enriched in GDM male foetal muscle around transcription starting sites. The rows showed the normalised unique tag counts for one H3K27ac binding event (ChIP peak) of the union, ordered by signalling strength; **B** H3K27ac occupancy at the *Ppargc1a* locus showed no significant difference between two groups determined by ChIP-seq analysis in foetal mouse skeletal muscle; **C** Schematic representation of selected loci for epigenetic experiments; **D** H3K4me3 ChIP-qPCR in foetal male skeletal muscle did not reveal difference in the promoter and enhancer regions of *Ppargc1α* between two groups (*n* = 5, multiple *t* tests); **E** DNA methylation of *Ppargc1α* at one CpG site showed no difference between groups (*n* = 6, two-tailed *t* test); **F** Representative western blotting images and analysis showed PGC1A, OXPHOS and pCREB were consistently down-regulated in GDM foetal muscle (*n* = 9, two-tailed *t* test); **G** ChIP-qPCR showed pCREB was fewer bound at the CRE element of *Ppargc1α*, *Pck* and *G6pc* in GDM foetal male skeletal muscle (n_CTR_= 4, n_GDM_ = 6, multiple *t* tests); **H** Heatmap of transcription level of *Pck* and *G6pc* showed they were both down-regulated in GDM foetal muscle according to RNA-seq (*n* = 5); **I** Representative mRNA levels of genes mentioned above in C2C12 were inhibited to some extent after CREB signalling inhibition (*n* = 4, multiple *t* tests); **J** Representative protein levels of CREB/PGC1A/OXPHOS signalling also demonstrated to be lower in C2C12 after CREB signalling inhibition (*n* = 3, two-tailed *t* tests); **K** Genes mentioned above were stimulated to transcript in C2C12 after CREB signalling stimulation (*n* = 3, multiple *t* tests). The data are expressed as the mean ± SEM. Significance of the differences: *p < 0.05, **p < 0.01, ***p < 0.001.

High glucose levels are thought to regulate gene expression via epigenetic modifications. According to the RNA sequencing results, the transcription of DNA methylases, such as *Dnmt1* and *Dnmt3b*, was evenly upregulated in GDM mice. However, the expression of the DNA demethylase *Tet1* was also induced in GDM groups. In addition, the transcription of the histone deacetylases *Hdac1*, *Hdac2,* and *Hdac6* was also induced (Supplementary Fig. 1N). Therefore, DNA methylation and histone modification of *Ppargc1α* might occur. We firstly performed H3K27ac ChIP sequence to assessed the abundance of H3K27ac in both groups (Fig. 4A), we found binding peak of H3K27ac was enriched in transcriptional starting sites (TSS) in GDM male foetus. Specifically, we took a look at the zoom detail of H3K27ac binding around *Ppargc1α* gene and found no significant difference (Fig. 4B). The binding of H3K4me3 in the promoter and enhancer regions of *Ppargc1α* between two groups also did not reveal difference (Fig. 4D). In addition, no difference in DNA methylation was found in the *Ppargc1α* differentially methylated region (DMR) (Fig. 4E). However, we found that the phosphorylation level of CREB was decreased in the SKM of GDM mice (Fig. 4F). Chromatin immunoprecipitation revealed fewer activated pCREB molecules in the binding sites of *Ppargc1α*, *Pck1* and *G6pc* (Fig. 4G), leading to decreased transcription of *G6pc* and *Pck1* in the RNA-seq profile (Fig. 4H). Further treatment with H89, the inhibitor of CREB signalling, dramatically decreased mitochondrial gene expression (Fig. 4I) and protein (Fig. 4J). Conversely, treatment with forskolin to activate CREB signalling significantly promoted the transcription of mitochondrial genes as well as *Ppargc1α* (Fig. 4K). In conclusion, we suggested that decreased phosphorylation of CREB in high glucose conditions decreases CREB binding to CRE elements in *Ppargc1α* genes, thus inhibiting its transcription and led to mitochondrial dysfunction in SKM of GDM male foetus.

### The F2 generation of GDM mice exhibited systematic metabolic disorders, muscle loss and mitochondrial abnormalities in the soleus

**Fig. 5:**
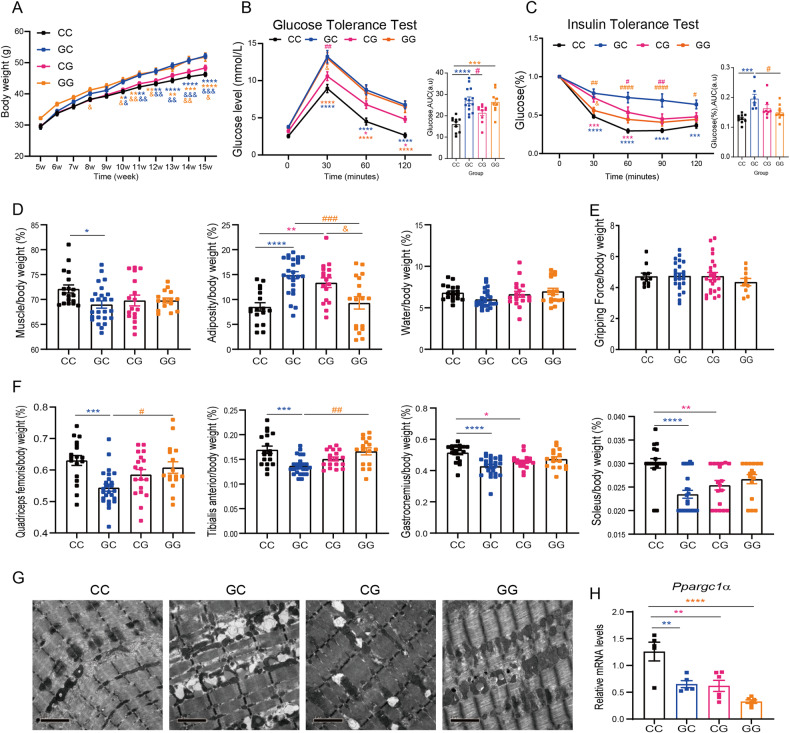
Intrauterine hyperglycaemia during late gestation caused metabolic disorders, sarcopenia, and mitochondrial abnormalities in the skeletal muscle of 16-week-old F2 males. **A** Growth curves of F2 male offspring showed GC and GG male were much heavier than others from 10 weeks old on (n_CC_= 21, n_GC_ = 41, n_CG_ = 34, n_GG_ = 20, 2-way ANOVA); **B** Evident impaired GTT was observed in the GC and GG male mice compared to CC, CG had higher glucose level at 60 and 120 min compared to CC (n_CC_= 10, n_GC_ = 14, n_CG_ = 10, n_GG_ = 9, 2-way ANOVA). AUC, area under the curve (ordinary one-way ANOVA); a.u., arbitrary units; **C** ITT line chart showed GC was mostly insulin resistant compared to other groups, CG had higher glucose level at 30 and 60 min compared to CC. AUC demonstrated GC male showed impaired ITT compared to CC and GG (n_CC_= 10, n_GC_ = 8, n_CG_ = 8, n_GG_ = 10, 2-way ANOVA, AUC, ordinary one-way ANOVA); **D** Less muscle was detected in GC, more fat was detected in GC and CG males in body composition analysis (n_CC_= 18, n_GC_ = 25, n_CG_ = 17, n_GG_ = 15, ordinary one-way ANOVA); **E** No difference was detected in gripping force among F2 generation (n_CC_= 11, n_GC_ = 24, n_CG_ = 24, n_GG_ = 10, ordinary one-way ANOVA); **F** Compared to CC male, four muscle weights in GC as well as GAS and soleus of CG males were significantly lighter (n_CC_= 18, n_GC_ = 25, n_CG_ = 18, n_GG_ = 17, ordinary one-way ANOVA); **G** TEM of SOL in the GC and CG groups exhibited severe mitochondrial abnormalities; **H** Quantification of mRNA levels of *Ppargc1α* in the soleus showed a decrease in F2 generation (n_CC_= 5, n_GC_ = 5, n_CG_ = 6, n_GG_ = 6, ordinary one-way ANOVA); Data are expressed as the mean ± SEM. Significance of the differences: *p < 0.05, **p < 0.01, ***p < 0.001, ****p < 0.0001 vs. CC; ^#^*p* < 0.05, ^##^*p* < 0.01, ^###^*p* < 0.001, ^####^*p* < 0.0001 vs. GC; ^&^*p* < 0.05, ^&&^*p* < 0.01 vs. CG.

We were curious about whether these effects could be inherited by generations. Interbreeding between 12-week-old F1 male and female mice from CTR and GDM groups was performed to obtain the second generation (F2), which could be divided into four groups: CTR♂×CTR♀ (CC), GDM♂×CTR♀ (GC), CTR♂×GDM♀ (CG) and GDM♂×GDM♀ (GG). We monitored body weight from 5 weeks to 15 weeks. Among the male mice, those in the GC and GG groups were heavier than others from ten weeks on (Fig. 5A), while among the females, those in the GC and CG groups were heavier than CC group (Supplementary Fig. 3A). We further performed GTTs and ITTs for both sexes at 16 weeks. Compared to those in the CC, evident impaired glucose tolerance was observed in the GC and GG male mice according to AUC-GTT. Interestingly, GC male mice also showed significant glucose intolerance compared to CG groups (Fig. 5B). Line chart revealed that GC and CG displayed insulin resistance to some extent compared to CC groups, but AUC demonstrated only GC male showed impaired systematic insulin resistance compared to CC and GG groups (Fig. 5C). Among F2 female, GC and CG showed higher glucose not only in GTT at the time point of 90 min but also in ITT at the time point of 120 min, but no notable significance was found in AUC of either GTT or ITT (Supplementary Fig. 3B, C). Body composition analyses revealed that GC males developed less muscle, more fat was detected in GC and CG males (Fig. 5D). However, the difference in grip strength between F2 males was not significant (Fig. 5E). The ratios of the four selected skeletal muscle weight ratios were all obviously lower in GC males. GAS and soleus of CG males were also significantly lighter than CC group (Fig. 5F). Moreover, GC and CG females also developed less muscle and more adiposity composition than CC female (Supplementary Fig. 3D). But, the ratio of QUA, GAS and soleus were only found to be less in GC group than CC groups (Supplementary Fig. 3E). Afterwards, we observed mitochondrial morphology in the soleus. TEM images suggested that the soleus of males (Fig. 5G) and females (Supplementary Fig. 3F) in the GC and CG groups exhibited severe mitochondrial abnormalities. We measured the mRNA level of *Ppargc1α* and detected a decrease in GDM-related F2 male offspring (Fig. 5H), but no significant change was found in F2 female offspring (Supplementary Fig. 3G).

## Discussion

In this study, we explored the effects of foetal exposure to short-term hyperglycaemia on muscle development and function in offspring mice. We demonstrated that gestational diabetes induced an intrauterine hyperglycaemic environment and caused glucose intolerance, sarcopenia, and exercise endurance capacity deficit in adulthood by inhibiting mitochondrial biogenesis and bioenergetics in foetal skeletal muscle. Our data suggested that high glucose suppressed the transcription of *Ppargc1α* in foetal GDM male muscle by inhibiting CREB phosphorylation and thus decreasing its binding to CRE elements of *Ppargc1α*, leading to abnormal mitochondrial structure and oxidative metabolism. In addition, we observed the intergenerational effects of gestational diabetes on F2. The F2 generation of GDM, especially GC group, exhibited obesity, glucose intolerance, and insulin resistance at 16 weeks. The muscles of GC male mice were lighter, the adiposity of GC and CG groups and the mitochondrial structure of these groups were also disrupted. Moreover, the transcription of *Ppargc1α* was still inhibited in the soleus of F2 generation of GDM male mice.

The results showed that foetal exposure to intrauterine hyperglycaemia during late gestation induced glucose intolerance and sarcopenia in GDM males (Fig. 1), which delayed and attenuated the appearance of the foetus in GDM females (Supplementary Fig. 2). Long-term exposure to intrauterine hyperglycaemia induced by STZ injection on the first day of pregnancy could generate offspring with obvious glucose intolerance [3, 5] at 8 weeks in both sexes. However, we did not find significant glucose insensitivity in 8-week-old female offspring. We attributed this discrepancy partially to the duration and time point of hyperglycaemic exposure. As reported, chronic high-fat dietary intervention before pregnancy did not contribute to an obvious metabolic disturbance in female offspring at 8 weeks as long as the mice maintained stabilised serum glucose during early pregnancy [14]. A sex-specific discrepancy was observed not only in systematic metabolism but also in skeletal muscle mass and mitochondrial structure in this study. We found that the skeletal muscle of GDM male offspring was lighter and that the levels of mitochondrial genes were generally lower at 8 weeks than those in the control group, while female offspring showed no differences. In addition, as shown by TEM, the mitochondrial structure was also destroyed in foetal female skeletal muscle (Supplementary Fig. 2H). There was also a decrease in mitochondrial oxygen consumption in myoblasts from female foetal limb muscles (Supplementary Fig. 2J). However, we did not detect significant changes in the expression of genes related to mitochondrial biogenesis or oxidative metabolism (Supplementary Fig. 2I). Normal mitochondrial structure and function are balanced by mitochondrial biogenesis, fission and fusion, mitophagy, and mtDNA copy number. We did not find significant changes in mitochondrial fusion or fission in foetal male muscle (Supplementary Fig. 1K), but we cannot exclude the possibility of altered mitochondrial fusion, fission or mitophagy in foetal female muscle. The mechanism through which intrauterine hyperglycaemia affects foetal skeletal muscle differs according to sex. Oestrogen was demonstrated to orchestrate metabolites in skeletal muscle [15]. Ovariectomized mice or mice with a deletion of oestrogen-related receptors demonstrated that oestrogen plays a protective role against muscle loss and mitochondrial organisation [16, 17] via oestrogen-related receptors α [18]. In another female mouse model, muscle-derived adipokines could maintain mitochondrial quality control by provoking mitochondrial fission and clearance [19]. In fact, in a protein restriction mouse model, sex determined the metabolic and molecular response to dietary intervention differently [20]. Integrated analysis of the transcriptome and methylome in human biopsies revealed that genes associated with muscle contraction and metabolism were strongly sex biased [21], which indicated that muscle may respond differently to the environment in males and females. In addition, gestational diabetic placenta displays sex-specific deregulation; for example, placenta in GDM mothers bearing a male foetus was more susceptible to hormone fluctuations [22].

Significant muscle loss was only found in QUA and TA of GDM males at 8 weeks (Fig. 1E), but skeletal muscle fatigability and mitochondrial dysfunction were demonstrated to be independent of muscle mass when interleukin-6 was chronically elevated [23]. Additionally, different muscle types can be influenced by different stimuli. For example, recent work has demonstrated that only glycolytic muscles, such as the extensor digitorum longus, rather than slow-twitch skeletal muscle, exhibit morphological and biochemical changes in male offspring with maternal vitamin D deficiency [24]. Poor insulin signalling reaction was once demonstrated in soleus muscle of 21-day-old weanlings exposed as foetus to hyperglycaemia [8], which means soleus can be first victim of elevated glucose exposure. In our work, we found mitochondrial malformation in SKM of GDM male was in accord with inhibited oxidative genes expression and poor-reacted insulin signalling pathway even at 8 weeks (Fig. 1F–J, Supplementary Fig. 1D–I). As mitochondria work as energy factories in cells, decreased mitochondrial content and impaired mitochondrial function can lead to insulin insensitivity via lipid deposition [25] and reactive oxygen species accumulation [26], inhibiting muscle renewal and increasing the chances of sarcopenia.

We verified that adverse mitochondrial structure and related genes were transcriptionally disrupted in foetal GDM male muscle (Fig. 2A, F, G). A high glucose concentration or diabetic environment was demonstrated to repattern mitochondrial function and energy metabolism in clinical trials [27–29] and cell culture [30–32]. According to the prediction by IPA, we confirmed that the regulator of mitochondrial biogenesis, *Ppargc1α* was restrained at the transcriptional level (Fig. 2L, N). The transcription of *Ppargc1α* can be regulated by epigenetic modulation or molecular signalling [33]. For example, the histone methyltransferase SMYD1 fuels cardiac mitochondrial metabolism by accumulating H3K4me3 at the *Ppargc1α* locus in mouse hearts [34]. Maternal succinate supplementation stimulates *Ppargc1α* expression by increasing H3K4me3 in the *Ppargc1α* promoter in foetal BAT [35]. 4-Phenylbutyric acid increased H3K27 acetylation at the *Ppargc1α* promoter and its expression [36]. Even though DNA methylation can regulate gene transcription, Barres, R et al. reported that DNA methylation of the *PPARGC1A* promoter region in human SKM showed no difference at either specific sites or average percentages although *PPARGC1A* gene expression in GDM offspring was lower than that in the background population [37]. In our work, although we found that the levels of key epigenetic enzymes (Supplementary Fig. 1N) were altered in the muscle of GDM foetuses, we did not detect significant H3K4me3 enrichment or DNA methylation around the *Ppargc1α* promoter or enhancer regions (Fig. 4D, E). ChIP sequence showed that peaks of H3K27ac seemed to enrich greatly in GDM around transcriptional starting sites among specific genes, but no significant enrichment was found around the *Ppargc1α* gene in GDM foetal muscle (Fig. 4B). Honestly, it was limited for us to pick H3K27ac and H3K4me3 for histone modification detection because other histone modifications, such as β-hydroxybutyrylation of H3K9 [38] and H4 acetylation [39], were also demonstrated to regulate the expression of *Ppargc1α*. Additionally, whether DNA methylation of *Ppargc1α* has been altered need to be explored further because we only selected specific CpG island in DMRs for detection, while in human samples, non-CpG methylation has been demonstrated to be negatively associated with the expression of PPARGC1A [28].

After further exploring the reasons for the decreased levels of *Ppargc1α* in the SKM of GDM offspring mice, we discovered that CREB phosphorylation (pCREB) was alleviated in high glucose conditions (Fig. 4F) and that the abundance of activated pCREB was reduced in the CRE elements of *Ppargc1α*, *Pck* and *G6pc* (Fig. 4G). An HDAC inhibitor (mocetinostat) activated the CREB/PGC1A signalling pathway to alleviate myocardial ischaemia/reperfusion injury [40], which was consistent with the high HDAC expression shown by our foetal RNA-seq data. Researchers have demonstrated that during fasting or in a low-glucose environment [41], activated CREB stimulates the hepatic expression of the *Pepck*, *G6pc* and *Ppargc1α* genes by binding to CRE within their promoters [42]. Feasible cAMP-CREB signalling is a key pathway involved in skeletal muscle metabolism and growth, which results from the transcriptional activation of genes that reduce muscle protein breakdown and increase mitochondrial biogenesis [43]. Our data revealed inactivated dephosphorylated CREB dissociated from the CRE element was effective at inhibiting *Ppargc1α* transcription in GDM foetal muscle (Fig. 4G, H), which may lead to the greater enrichment of H3K27ac in the GDM foetal muscle genome (Fig. 4A). Similarly, high salt concentrations increase H3K27ac on the *Sirtuin 3* promoter, thus inhibited the binding of another transcription regulator, nuclear factor erythroid 2-related Factor 2 (*Nrf2*) and resulting in sustained inhibition of *Sirtuin 3* expression [44].

Further effects of intrauterine hyperglycaemia during late gestation were observed in the F2 generation (Fig. 5). We found that GC and GG males developed obesity and glucose intolerance. Muscle mass and mitochondrial morphology both exhibited adverse changes in mainly GC and CG groups. Recently, researchers reported that acquired DNA methylation at CpG islands in embryonic stem cells or mice can be transgenerationally inherited [45]. Once exposed to a hyperglycaemic environment, primordial germ cells carry epigenetic memories to the next generation [46]. Our previous work demonstrated that maternal diabetes increased birth weight in F2 offspring with impaired glucose tolerance through the paternal line [3]. Another article by our team revealed that diabetic mothers exhibited maternal inheritance of glucose intolerance via oocyte *Tet3* insufficiency [47]. Therefore, in our F2 model, we attributed the adverse phenotype to hypermethylation in sperm compared to that in CC. With respect to the CG, we attributed this to the imbalance in DNA methylase and demethylase expression in the oocytes of GDM females.

## Conclusions

Intrauterine hyperglycaemia during late gestation suppressed mitochondrial biogenesis and oxidative metabolism in foetal skeletal muscle through CREB/PGC1A signalling, leading to sarcopenia, metabolic disorder and exercise endurance capacity deficit in male adulthood. Glucose intolerance, muscle loss and mitochondrial abnormalities were also observed in GDM-F2, especially in GC and CG groups. Decreased transcription of *Ppargc1α* in the soleus might be inherited by the F2 generation through aberrant DNA methylation of gamete.

### Supplementary information

supplementary figure 1

supplementary figure 2

supplementary figure 3

supplementary figures 1-3 and supplementary tables 1-2

Data Set 1

Data Set 2

Data Set 3

## Data Availability

We have submitted RNA sequence data to the GEO repository (NCBI GEO accession number: GSE255246) and proteomics data to the Proteomics Identification database (Private Project PXD049987). Before the official release, datasets will be available from the corresponding author upon reasonable request.
