# Supplementary figures and images for: Intrauterine hyperglycaemia during late gestation caused mitochondrial dysfunction in skeletal muscle of male offspring through CREB/PGC1A signaling
Source: Nutr Diabetes. 2024 Jul 23;14:56. doi: 10.1038/s41387-024-00299-x (PMC11266655; doi:10.1038/s41387-024-00299-x)

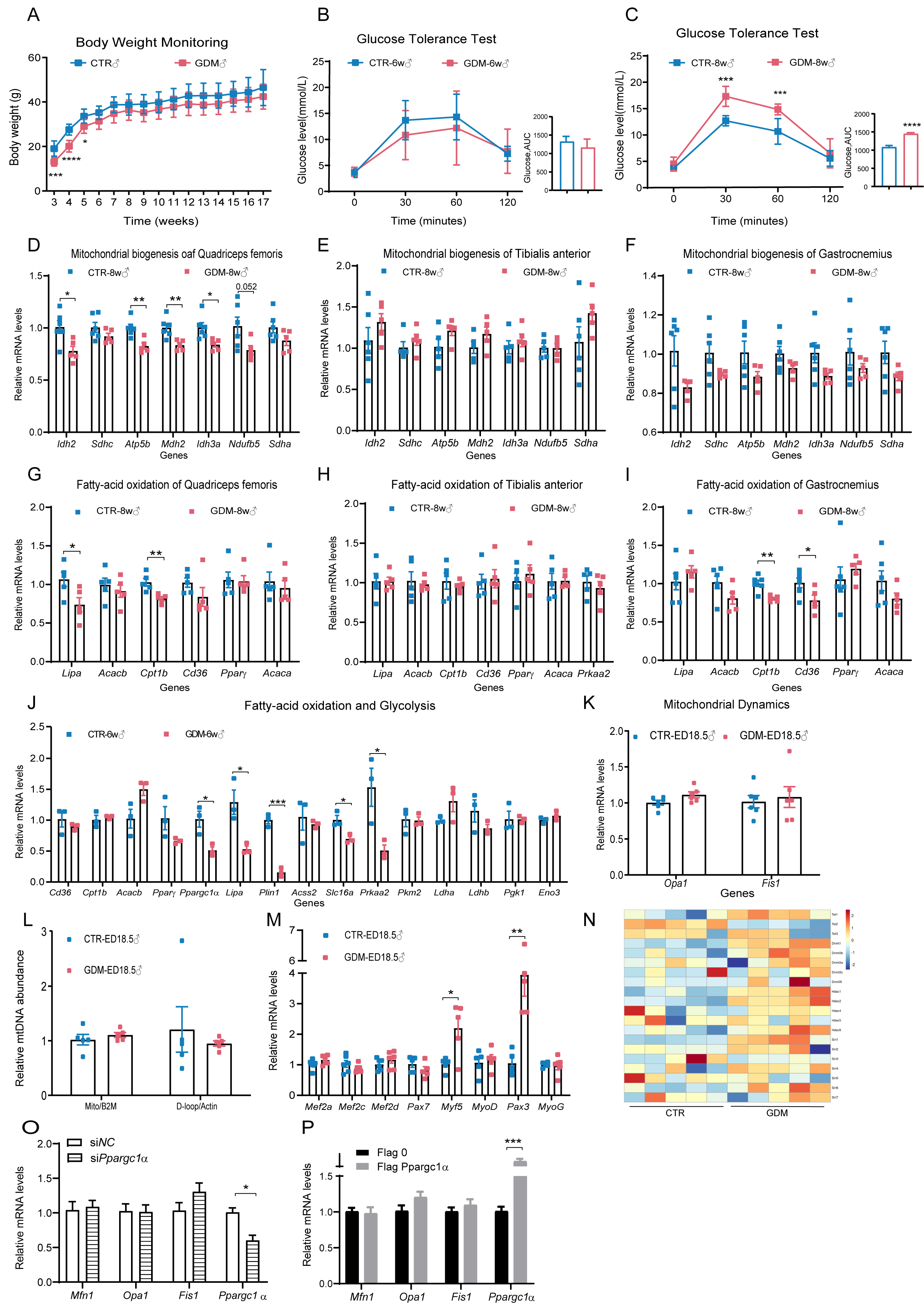

Supplement: Supplementary file 1 — supplementary figure 1 [file 41387_2024_299_MOESM1_ESM.tif]

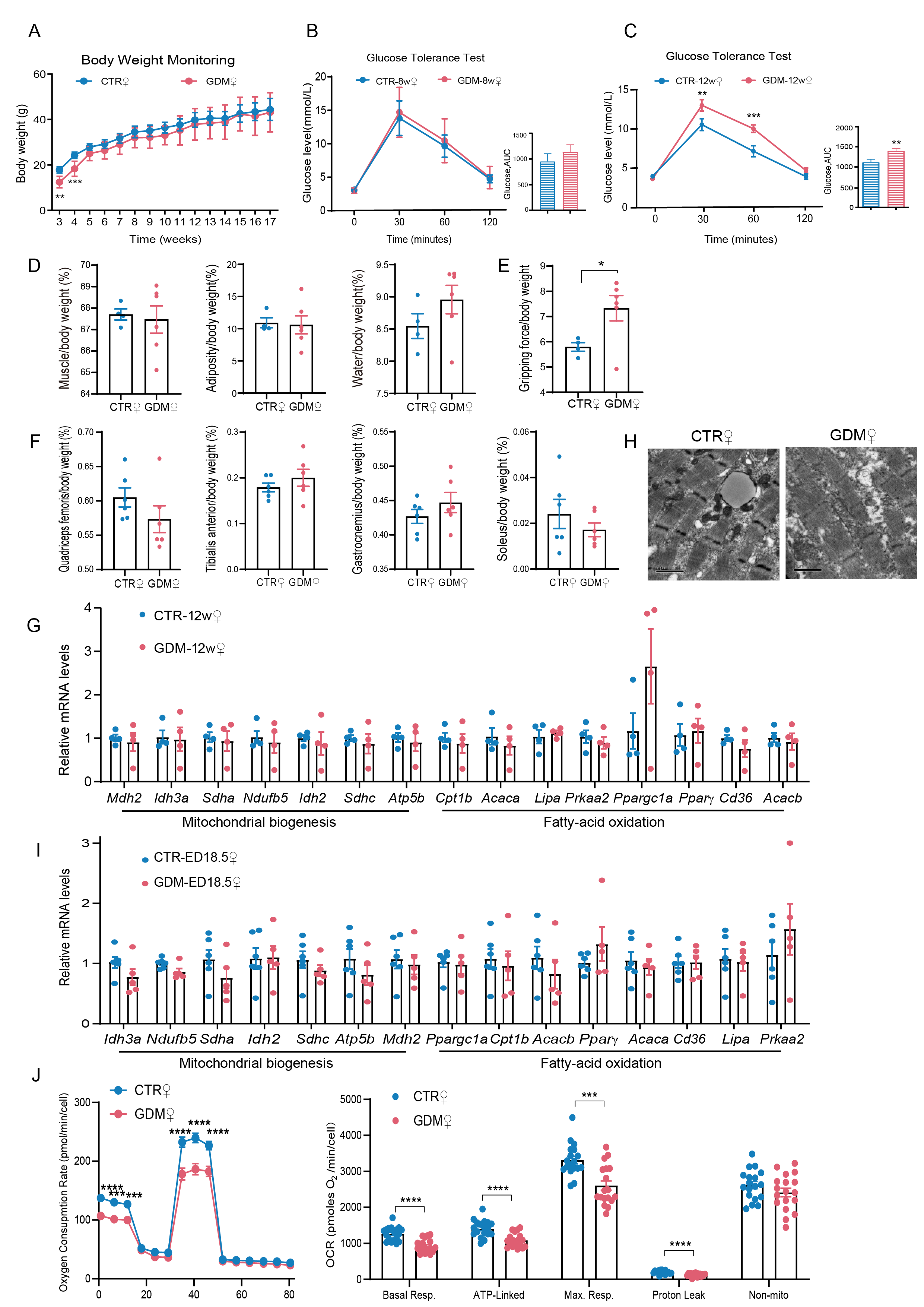

Supplement: Supplementary file 2 — supplementary figure 2 [file 41387_2024_299_MOESM2_ESM.tif]

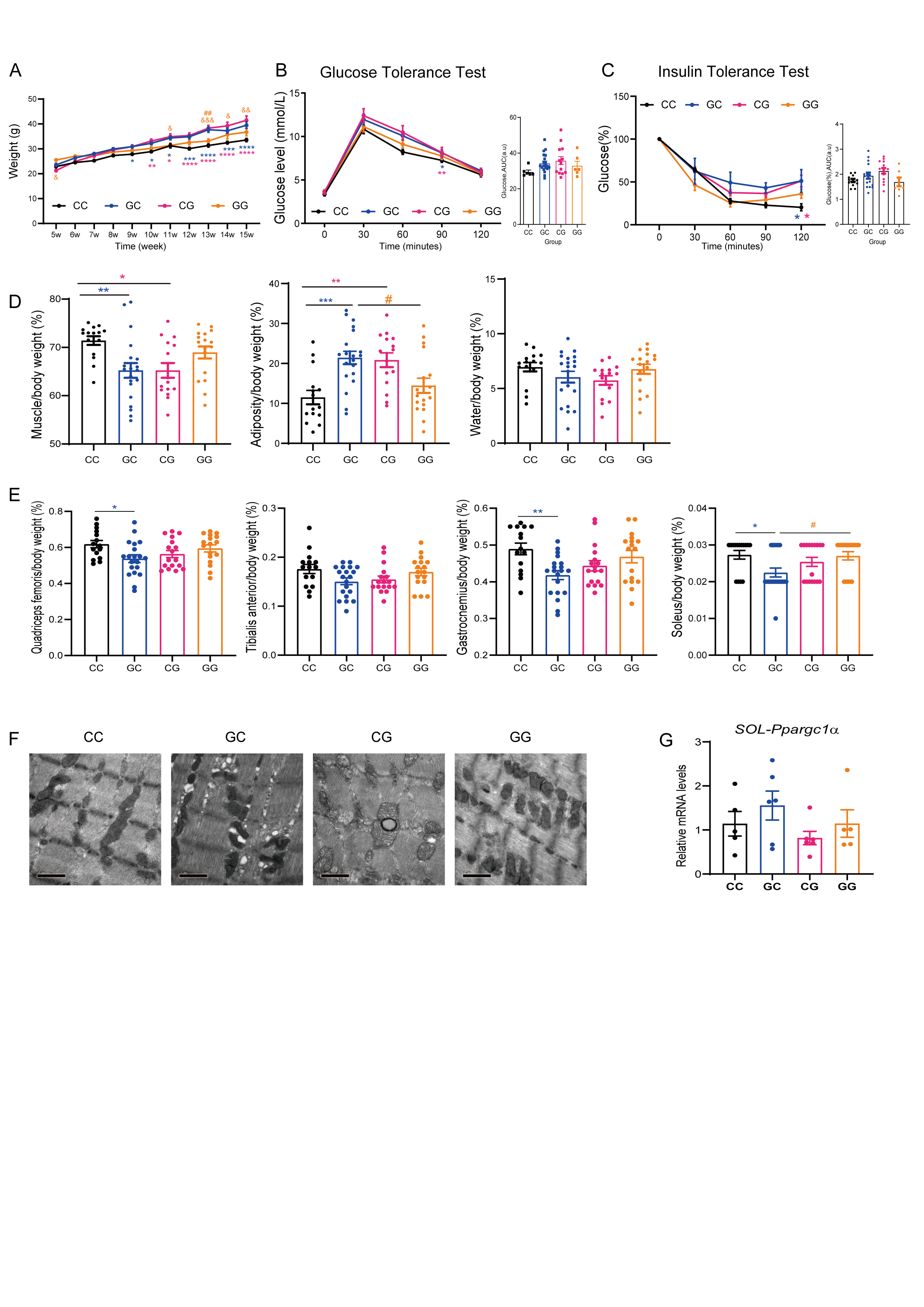

Supplement: Supplementary file 3 — supplementary figure 3 [file 41387_2024_299_MOESM3_ESM.tif]
