# Supplementary material for: Intrauterine hyperglycaemia during late gestation caused mitochondrial dysfunction in skeletal muscle of male offspring through CREB/PGC1A signaling
Source: Nutr Diabetes. 2024 Jul 23;14:56. doi: 10.1038/s41387-024-00299-x (PMC11266655; doi:10.1038/s41387-024-00299-x)
Supplement: Supplementary file 4 — supplementary figures 1-3 and supplementary tables 1-2 [file 41387_2024_299_MOESM4_ESM.docx]

**Supplementary Figure 1**

A: Growth curves of F1 male showed body weight was significantly lower in GDM offspring before 5 weeks old (n_CTR_=13, n_GDM_=16, 2-way ANOVA); B: No difference in GTT was found between two groups in 6-week-old F1 male (n=6, 2-way ANOVA, AUC, two-tailed t test); C: Significant difference in GTT was found in 8-week-old GDM male (n=6, 2-way ANOVA, AUC, two-tailed t test); (D-F): Representative mRNA levels of genes related to mitochondrial biogenesis showed somewhat decreases in QUA(D), but not in TA(E) or GAS (F) of 8-week-old GDM male (n_CTR_=6, n_GDM_=5, multiple t tests); (G-I): Representative mRNA levels of genes related to fatty-acid oxidation was inhibited to some extent in QUA(G) and GAS (I) but not in TA(H) of 8-week-old GDM male (n_CTR_=6, n_GDM_=5, multiple t tests); J: Transcription of oxidation and glycolysis had a deficit in 6-week-old GDM male (n=3, multiple t tests); K-M: Mitochondrial dynamics (K, n=6), copy numbers(L, n=5) and muscle development-related genes (M, n=5, multiple t tests) in F1 foetal skeletal muscle show no difference between groups at all; N: Heatmap of genes related to epigenetic enzymes in F1 foetal skeletal muscle according to RNA-seq; O-P: The mRNA levels of mitochondrial fusion and fission did not show difference after knocking out (O, n=6, multiple t tests) or overexpressing *Ppargc1α* (P, n=6, multiple t tests). Data are expressed as mean±SEM. Significance of the differences: *p<0.05, **p<0.01, ***p<0.001.

**Supplementary Figure 2**

A: Growth curves of F1 female showed body weight was significantly lower in GDM offspring before 4 weeks old (n_CTR_=15, n_GDM_=8, 2-way ANOVA); B: No difference in GTT was found between two groups in 8-week-old F1 female (n=4, 2-way ANOVA, AUC, two-tailed t test); C: Significant difference in GTT was found in 12-week-old GDM female (n=17, 2-way ANOVA, AUC, two-tailed t test); D: Body composition analysis of F1 12-week-old female mice showed no dramatic difference (n_CTR_=4, n_GDM_=6, two-tailed t test); E: Gripping force normalized to body weight was much bigger in F1 female at 12 weeks (n_CTR_=4, n_GDM_=6, two-tailed t test); F: QUA, TA, GAS and SOL weights normalized to body weight showed no difference in F1 female(n=6, two-tailed t test); G: Representative mRNA levels of mitochondrial biogenesis and fatty-acid oxidation did not show obvious discrepancy in F1 female soleus muscle at 12 weeks (n=4, multiple t tests); H: TEM displayed disrupted mitochondrial structure in foetal muscle of GDM female; I: Representative mRNA levels of mitochondrial biogenesis and fatty-acid oxidation show no obvious discrepancy in foetal female skeletal muscle (n_CTR_=6, n_GDM_=5, multiple t tests); J: OCR was decreased in GDM female myoblast by 2-way ANOVA. Among them, only OCR in mitochondria was decreased (n=8, each “n” refers to a single replicate, multiple t tests). Data are expressed as mean±SEM. Significance of the differences: **p<0.01, ***p<0.001.

**Supplementary Figure 3**

A: Growth curves of F2 female offspring showed GC and CG groups were heavier than CC group from 10 weeks old on (n_CC_=14, n_GC_=20, n_CG_=15, n_GG_=9, 2-way ANOVA); B: GTT showed glucose levels were higher in GC and CG at the time point of 90 minute, but AUC did not reveal significant systematic difference among F2 female (n_CC_=6, n_GC_=20, n_CG_=13, n_GG_=6, 2-way ANOVA, AUC, ordinary one-way ANOVA); C: ITT showed glucose levels were higher in GC and CG at the time point of 120 minute, but AUC did reveal no difference (n_CC_=12, n_GC_=16, n_CG_=13, n_GG_=7, 2-way ANOVA, AUC, ordinary one-way ANOVA); D: Less muscle and more fat were detected in GC and CG females in body composition analysis (n_CC_=15, n_GC_=20, n_CG_=15, n_GG_=17, ordinary one-way ANOVA); E: QUA, GAS and SOL weights in GC female were obviously lighter than CC (n_CC_=15, n_GC_=20, n_CG_=17, n_GG_=17, ordinary one-way ANOVA); F: TEM of SOL in GC and CG female exhibited mitochondrial abnormalities; G: Quantification of mRNA levels of *Ppargc1α* in soleus among F2 female did not show significant difference (n_CC_=5, n_GC_=6, n_CG_=6, n_GG_=5, ordinary one-way ANOVA); Data are expressed as mean±SEM. Significance of the differences: *p<0.05, **p<0.01, ***p<0.001, ****p<0.0001 vs CC; #p<0.05, ##p<0.01 vs GC; &p<0.05, &&p<0.01, &&&<0.001 vs CG.

**Supplementary Table 1.** Antibodies used for western blot

| **Primary antibody** | **RRID** | **Identifier** | **Source** | **Dilution** |
| --- | --- | --- | --- | --- |
| anti-AKT | AB_915783 | 4691S | Cell  Signaling  Technology | 1:2000 |
| anti-pAKT (S473) | AB_2315049 | 4060S |  | 1:2000 |
| anti-S6 | AB_331355 | 2217S |  | 1:10000 |
| anti-pS6 (S235/236) | AB_916156 | 4858S |  | 1:10000 |
| anti-CREB | AB_331277 | 9197 |  | 1:2000 |
| anti-pCREB(S133) | AB_2561044 | 9198S |  | 1:2000 |
| PGC1A | AB_2890187 | Sc-518025 | SANTA CRUZ | 1:1000 |
| PGC1A | AB_881987 | Ab54481 | Abcam | 1:1000 |
| GAPDH | AB_2630358 | Ab181602 |  | 1:10000 |
| OXPHOS cocktail | AB_2629281 | Ab110413 |  | 1:200 |
| H3K27ac | AB_2118291 | Ab4729 |  | 1.5ml for each |
| H3K4me3 | AB_306649 | Ab8580 |  | 1.5ml for each |
| Rat-anti-laminin | AB_477153 | #L0663 | Sigma | 1:1000 |
| Myosin Heavy Chain Type Ⅰ | AB_2235587 | BA-D5 | DSHB | 1:100 |
| Myosin Heavy Chain Type Ⅱa | AB_2147165 | SC-71 |  | 1:100 |
| Myosin Heavy Chain Type Ⅱb | AB_2266724 | BF-F3 |  | 1:100 |
| **Secondary antibody** |  | **Identifier** | **Source** | **Dilution** |
| Goat anti-mouse IgG1-Alexa Fluor-488 | AB_2338854 | 115-545-205 |  | 1:500 |

| Goat anti-mouse IgG2b-DyLight-405 | AB_2338801 | 115-475-207 | Jackson ImmunoResearch | 1:500 |
| --- | --- | --- | --- | --- |
| Goat anti-mouse IgM-Alexa Fluor-594 | AB_2338880 | 115-585-075 |  | 1:500 |

| Goat anti-rat IgG-Alexa Fluor-647 | AB_141778 | #A-21247 | Thermo Fisher Scientific | 1:500 |
| --- | --- | --- | --- | --- |
| Goat anti-mouse H&L (HRP) | AB_955439 | Ab6789 | Abcam | 1:20000 |
| Goat anti-rabbit H&L (HRP) | AB_955447 | Ab6721 |  | 1:20000 |

**Supplementary Table 2.** Oligonucleotide primers used for qPCR and DNA methylation analysis

| **Genes** | **Forward** | **Reverse** |
| --- | --- | --- |
| *β actin* | AGTGTGACGTTGACATCCGT | GCAGCTCAGTAACAGTCCGC |
| *Cd36* | TTAGATGTGGAACCCATAACTGGA | TTGACCAATATGTTGACCTGCAG |
| *Acaca* | TAATGGGCTGCTTCTGTGACTC | CTCAATATCGCCATCAGTCTTG |
| *Acacb* | CCGCTCACCAACAGTAAGGT | ATGAGCTTGGCAGGGAGTTC |
| *Pparγ* | CCGTAGAAGCCGTGCAAGAG | GGAGGCCAGCATCGTGTAGA |
| *Lipa* | GACCACTCCCGATGCAACTC | GACCGAGTGTTCCTCACCAG |
| *Prkaa2* | GGCAAAGTGAAGACTACCAGG | CTTCAACCCGCCCATGTTTG |
| *Cpt1b* | CATGTATCGCCGCAAACTGG | CCTGGGATGCGTGTAGTGTT |
| *Mdh2* | GACCTGTTCAACACCAACGC | GACCTGTTCAACACCAACGC |
| *Ndufb5* | CTTCGAACTTCCTGCTCCTT | GGCCCTGAAAAGAACTACG |
| *Sdha* | GGAACACTCCAAAAACAGACCT | CCACCACTGGGTATTGAGTAGAA |
| *Sdhc* | GCTGCGTTCTTGCTGAGACA | ATCTCCTCCTTAGCTGTGGTT |
| *Cox5b* | AAGTGCATCTGCTTGTCTCG | GTCTTCCTTGGTGCCTGAAG |
| *Atp5b* | GGTTCATCCTGCCAGAGACTA | AATCCCTCATCGAACTGGACG |
| *Idh2* | GGAGAAGCCGGTAGTGGAGAT | GGTCTGGTCACGGTTTGGAA |
| *Idh3a* | CCCATCCCAGTTTGATGTTC | ACCGATTCAAAGATGGCAAC |
| *Ppargc1α* | AGCCGTGACCACTGACAACGAG | GCTGCATGGTTCTGAGTGCTAAG |
| *Ppargc1β* | TCCAGAAGTCAGCGGCCTTGTGTCA | CTCTGGGACAGGGCAGCACCGA |
| *Plin1* | CTGTCTGAGACTGAGGTGGC | TCAGGGAGGTCTCCATCCAG |
| *Pkm2* | ATCATTTGTACCATTGGGCCTG | TTCATTCCAGACTTAATCATCTCCTTC |
| *Ldha* | TGCCTACGAGGTGATCAAGCT | GCACCCGCCTAAGGTTCTTC |
| *Ldhb* | AGTCTCCCGTGCATCCTCAA | AGGGTGTCCGCACTCTTCCT |
| *Hk2* | CCGCCGTGGTGGACAAGATA | AGCAGTGATGAGAGCCGCTC |
| *Eno3* | TGAACTGACACTGTCCCAGC | GTGAGTGAGCAAGAGTGGACA |
| *Opa1* | CTAAACCATTGTAACCTTTGTC | CTCTAATCGCCTAACTTCAG |
| *Fis1* | GCTGGTGTCTGTGGAGGACC | GCAGCACGATGCCTTTACG |
| *B2M* | ATGGGAAGCCGAACATACTG | CAGTCTCAGTGGGGGTGAAT |
| *Mito* | CTAGAAACCCCGAAACCAAA | CCAGCTATCACCAAGCTCGT |
| *D-loop* | TCACCCTATTAACCACTCA | A GACAGATACTGCGACATA |
| *Mef2a* | TAAAATCGCACCTGGCTTGC | GCTAATGTTGAGCTGGCTGC |
| *Mef2c* | GCTTCAATACTGCCAGTGCG | GTGGTACGGTCTCCCAACTG |
| *Mef2d* | TCTCTGGCACTAAGGACCCC | GAATGTCACCTGGCGGTTCC |
| *Pax7* | TGGGGTCTTCATCAACGGTC | ATCGGCACAGAATCTTGGAGA |
| *Pax3* | CCTCTGCCCAACCATATCCG | ATTTCCCAGCTAAACATGCCC |
| *Myf5* | TGCCAGTTCTCCCCTTCTGA | AGGCTGCTACTCTTGGCTCA |
| *MyoD* | CCACTCCGGGACATAGACTTG | AAAAGCGCAGGTCTGGTGAG |
| *MyoG* | GCAGGCTCAAGAAAGTGAATGA | TAGGCGCTCAATGTACTGGAT |
| **H3K4me3 binding sites** | | |
| *Site1* | CAGGAATGATTCCCAGAGCCACAG | CAGCGTCCAGCCTTAGATTGAGTC |
| *Site2* | CAGCGTCCAGCCTTAGATTGAGTC | AATTGTTGGAGTAGCCAGCGTAACC |
| *Site3* | GTATCACTGCACCACACGGACATC | ATTGAATTGCGCGGAGGATCGG |
| *Site4* | TGCAAACACGCTGAAGTCCTCTG | CAATTCCACAGTCTCTGCTGGCTAC |
| *Site5* | CAAGCCATCCAGCTCCCGAATG | CAGGTGCCTTCAGTTCACTCTCAG |
| **CRE element for CREB binding** | | |
| *Ppargc1α* | CAGAGGGCTGCCTTGGA | CAGCCTCCCTTCTCCTGTG |
| *G6pc* | TGTGCCTGTTTTGCTATTTTACG | AAGGTGCATCATCAGTAGGTTGA |
| *Pck* | CCCTGGAGTTTATTGTGTTAAGTCAGT | GCAGGCCTTTGGATCATAGC |
| **DNA methylation analysis** | | |
| *Ppargc1*α | GGGTGTAGTTATTGTGTTAGTAATAGG | Biotin-TCCAAAACAACCCTCTACCTC |
| Sequence: TGTAGGAGATTTGAGTTATTA | | |
